# Bioactive Peptides from *Meretrix lusoria* Enzymatic Hydrolysate as a Potential Treatment for Obesity in *db/db* Mice

**DOI:** 10.3390/nu16121913

**Published:** 2024-06-17

**Authors:** Ramakrishna Chilakala, Hyeon Jeong Moon, Min Seouk Jung, Jong Won Han, Kang Ho Ko, Dong Sung Lee, Sun Hee Cheong

**Affiliations:** 1 Department of Marine Bio-Food Sciences, Chonnam National University, Yeosu 59626, Republic of Korea; ramach2006@gmail.com (R.C.); dals404@naver.com (H.J.M.); erfg6203@naver.com (M.S.J.); hyssop4@gmail.com (J.W.H.); rkdghrh@naver.com (K.H.K.); 2 College of Pharmacy, Chosun University, Dong-gu, Gwangju 61452, Republic of Korea; dslee2771@chosun.ac.kr

**Keywords:** bioactive peptides, obesity, *db/db* mice, enzymatic hydrolysate, AMPK pathway

## Abstract

Obesity is acknowledged as a significant risk factor for cardiovascular disease, often accompanied by increased inflammation and diabetes. Bioactive peptides derived from marine animal proteins show promise as safe and effective anti-obesity agents by regulating adipocyte differentiation through the AMPK signaling pathway. Therefore, this study aims to investigate the anti-obesity and anti-diabetic effects of bioactive compounds derived from a *Meretrix lusoria* Protamex enzymatic hydrolysate (MLP) fraction (≤1 kDa) through a 6-week treatment (150 mg/kg or 300 mg/kg, administered once daily) in leptin receptor-deficient *db/db* mice. The MLP treatment significantly decreased the body weight, serum total cholesterol, triglycerides, and LDL-cholesterol levels while also exhibiting a beneficial effect on hepatic and serum marker parameters in *db/db* mice. A histological analysis revealed a reduction in hepatic steatosis and epididymal fat following MLP treatment. Furthermore, poor glucose tolerance was improved, and hepatic antioxidant enzyme activities were elevated in MLP-treated mice compared to *db/db* control mice. Western blot analysis showed an increased expression of the AMPK protein after MLP treatment. In addition, the expression of lipogenic genes decreased in *db/db* mice. These findings indicate that bioactive peptides, which are known to regulate blood glucose levels, lipid metabolism, and adipogenesis, could be beneficial functional food additives and pharmaceuticals.

## 1. Introduction

Obesity is one of the most significant health problems worldwide today, and its prevalence, along with related illnesses, is continually rising [1]. This chronic condition is characterized by excessive body fat accumulation and is associated with a shortened life expectancy. Furthermore, it significantly increases the risk of several diseases, such as cardiovascular disease (CVD), type 2 diabetes mellitus (T2DM), and nonalcoholic fatty liver disease (NAFLD) [2]. The intake of more energy in the body than is expended often leads to obesity, a process that can be influenced by hereditary, physiological, and/or environmental factors [3,4]. It is a major risk factor for T2DM, insulin resistance, and other metabolic syndromes. Insulin resistance and prediabetes typically lead to an increase in the number of islet β-cells via the proliferation of the pre-existing cellular population. Furthermore, increased insulin production occurs before diabetes onset to compensate for decreased hormone action in peripheral tissues (including the liver and skeletal muscle). The clinical onset of type 2 diabetes (T2DM) occurs when a decline in islet β-cell insulin output and/or a reduction in the β-cell number exists [5]. The main approaches to treating obesity include diet control, exercise therapy, medication therapy, and surgical therapy [6,7]. However, the efficacy of these approaches is not satisfactory. Moreover, unfavorable side effects restrict the use of drugs, including orlistat, bupropion/naltrexone, lorcaserin, phentermine/topiramate, liraglutide (3.0 mg), diethylpropion, and other drugs [2,8]. Given these challenges, developing innovative, safe, long-lasting, and widely accessible obesity treatments is crucial.

Bioactive peptides are naturally occurring compounds that have gained interest as a promising area of study owing to their potential antioxidant, anti-obesity, and anti-diabetic properties, as well as their diverse modes of action. Bioactive peptides are short amino acid sequences derived from dietary proteins [9,10,11]. They facilitate targeting and interactions with biological processes and receptors associated with lipid metabolism, appetite regulation, and adipogenesis. These bioactive peptides precisely modulate pathways related to obesity, effectively regulating energy balance and weight management [12]. Recent studies suggest that bioactive peptides derived from various marine proteins, including *Scapharca subcrenata* [13], tuna skin collagen [14], *Mustelus mustelus* [15], *Gadus chalcogrammus* [16], *Ruditapes philippinarum* [17], and *Meretrix lusoria* [18], demonstrate anti-obesity and anti-diabetic effects based on their peptide sequence, length, and composition [19]. Bioactive peptides, specifically enzymatic hydrolysates <1 kDa, demonstrate potent anti-obesity effects by reducing food consumption and body weight (BW). The management of hunger likely contributes to weight loss. These peptides promote lipolysis and prevent intracellular lipid accumulation. Additionally, they downregulate adipogenic transcription factors, such as peroxisome proliferator-activated receptor (PPAR-γ), CCAAT/enhancer binding protein alpha (C/EBP-α), and sterol regulatory element binding protein (SREBP-1), as well as acetyl-CoA carboxylase (ACC) and fatty acid synthase (FAS) protein expressions via modulating multiple underlying molecular mechanisms in obese mice [20,21]. Energy homeostasis is regulated by the enzyme adenosine monophosphate-activated protein kinase (AMPK), which is essential in preventing obesity, as it regulates fatty acid oxidation, glucose transport, hepatic lipid metabolism, and adipocyte lipolysis [22].

*Meretrix lusoria* (ML)—commonly known as the Asian hard clam—is distributed around Korea, Japan, and China [23]. The enzymatically hydrolyzed extract of ML meat contains bioactive peptides with antihypertensive, hypolipidemic, and antioxidant effects [18,24]. ML and its extracts are widely employed as traditional Chinese therapies for liver disease and chronic hepatitis, and they possess antitumor properties. Additionally, ML hot-water extract is marketed in Taiwan as a nutraceutical [25,26]. Moreover, it is an excellent source of several nutrients, such as polysaccharides, minerals, proteins, bioactive peptides, essential amino acids, and vitamins, all of which can benefit consumer health [27]. Therefore, this study aimed to investigate the effects of MLP hydrolysate on type 2 diabetes and obesity, considering the typical use of ML. In this investigation, MLP was produced through enzymatic digestion, and peptide components were identified via LC-MS/MS analysis. Subsequently, the antioxidant activity of the MLP hydrolysate fractions was evaluated. Furthermore, we demonstrated that MLP may significantly lower gene expressions associated with diabetes and obesity using *db/db* mice. It also exerts its hypoglycemic and anti-obesity effects by modulating the AMPK pathway. Our research identifies the mechanisms by which MLP hydrolysate inhibits obesity and diabetes, suggesting that MLP can be used as a natural product for treating these conditions.

## 2. Materials and Methods

### 2.1. MLP Enzymatic Hydrolysate Preparation

ML was obtained from a fish market in Yeosu, South Korea, and enzymatic hydrolysate was prepared using Protamex. First, 50 g of freeze-dried ML powder was combined with 0.5 g of Protamex enzyme, and the pH was adjusted to 6.0 at a temperature of 60 °C. The hydrolysis process was conducted for 24 h with continuous stirring. Following that, the entire mixture was heated to 100 °C for 10 min in a water bath to inactivate the enzymes. Using a filter cloth, unhydrolyzed protein molecules were then removed via filtration. The extraction yield was calculated by dividing the weight of the enzymatic hydrolysate by the percentage of dried weight of ML protein content used for the extraction. Following a previously established method [28], the filtrate sample was fractionated using an ultrafiltration membrane through the GE Healthcare Quixstand^TM^ benchtop system (Little Chalfont, Buckinghamshire, UK). This process led to four fractions: ≤1 kDa, 1–3 kDa, 3–5 kDa, and ≥5 kDa. Following freeze drying, the MLP ≤ 1 kDa fraction was chosen for the subsequent analysis.

### 2.2. MLP Antioxidant Activity and Reducing Power

The scavenging effect of MLP hydrolysate on DPPH, ABTS, and hydroxyl free radicals, including its ferric-reducing power, was analyzed in vitro at concentrations of 0.25, 0.5, 1, and 2 mg/mL. For DPPH radical scavenging [29], 50 µL of MLP hydrolysate at each concentration was mixed with a methanolic DPPH (0.208 mM, #1898664, Sigma-Aldrich, St. Louis, MO, USA) solution. The mixture was allowed to react for 20 min at room temperature in the dark. Using a microplate reader (Multiskan SkyHigh, Thermo Scientific, Seoul, Republic of Korea), the absorbance was measured at 540 nm. The DPPH scavenging activity (%) was calculated as [(A_Con_ − A_Sample_)/A_Con_] × 100.

According to Zhao et al. [30], ABTS radicals were produced by mixing a 7 mM ABTS (#30931670, Sigma-Aldrich, St. Louis, MO, USA) solution with 140 mM of potassium persulfate and allowing the mixture to stand in the dark at room temperature for 16 h. Before use, this mixture was diluted with 0.2 M of phosphate-buffered saline (pH 7.4). After mixing 100 µL of the diluted ABTS reagent with 100 µL of concentrated MLP hydrolysate, the mixture was left to stand at room temperature for 6 min. The absorbance was measured at 734 nm. A blank sample consisting of 200 µL of distilled water was utilized as a reference. The ABTS scavenging activity (%) was calculated using the following formula: [(A_Blank_ − A_Sample_)/A_Blank_] × 100.

To assess the hydroxyl radical scavenging activity [31], 1 mL of 6 mM FeSO_4_ solution (#7782630, Sigma-Aldrich, St. Louis, MO, USA) was mixed with 1 mL of MLP hydrolysate and 1 mL of 6 mM H_2_O_2_ solution. After shaking, the mixture was incubated at room temperature for 10 min. Following this, 1 mL of 6 mM salicylic acid was added, and the absorbance at 510 nm was measured 30 min later. A blank sample was prepared using 1 mL of distilled water instead of using the sample. The OH radical scavenging activity (%) was calculated using the formula [1 − (A_Sample_ − A_0_)/A_Blank_] × 100, where A_0_ represents the sample absorbance without salicylic acid. Using the radical scavenging activity relationship curve with sample concentrations, the half-inhibitory (IC_50_) values were also computed.

To perform the ferric-reducing power analysis [27], 100 μL of MLP hydrolysate and 100 μL of 0.2 M sodium phosphate buffer (pH 6.6) were combined. Subsequently, 100 μL of 10% potassium ferricyanide (#13746662, Sigma-Aldrich, St. Louis, MO, USA) was added. After incubating the mixture for 20 min at 50 °C in a dark area, 100 μL of 10% trichloroacetic acid was added to the mixture, which was then centrifuged for 10 min at 1000× *g* to precipitate it. The supernatant was mixed with 0.1% ferric chloride at a 5:1 ratio, and the absorbance was subsequently measured at 700 nm.

### 2.3. Cell Culture and Viability Assay

The L6 myoblast cell line, obtained from rat skeletal muscle (Manassas, VA, USA; ATCC^®^ number: CRL-1458), was cultured at 37 °C in a humidified environment with 5% CO_2_ using DMEM supplemented with 10% (*v*/*v*) heat-inactivated FBS, penicillin G (100 U/mL), and streptomycin (100 μg/mL), as previously mentioned (Kim et al., 2020). The effect of the MLP hydrolysate fraction (≤1 kDa) on cell viability was evaluated by measuring mitochondrial reductase function. This is based on the principle that tetrazolium salt 3-[4,5-dimethylthiazol-2-yl]-2,5-diphenyltetrazolium bromide (MTT) is reduced to formazan crystals. Each cell suspension (1 × 10^5^ cell/mL per well of the 96-well plates) was mixed with 5 mg/mL of MTT for 4 h in order to determine the viability of the cells. Following the dissolution of the formazan crystals in the cells in DMSO, the optical density of each sample group solution was verified at a wavelength of 540 nm.

### 2.4. Peptide Characterization via LC-MS/MS

A Sephadex G-25 gel filtration column (2.5 × 70 cm) (GE Healthcare, Chicago, IL, USA) was employed to separate sub-fractions following the dissolution of the MLP hydrolysate fraction (≤1 kDa) in distilled water (DW). Following column elution with DW at a flow rate of 1.5 mL/min, four sub-fractions were collected. The scavenging ability of these MLP sub-fractions against DPPH radicals was assessed. MLP hydrolysate sub-fraction samples were analyzed at wavelengths of 254 and 280 nm by injecting 20 μL into a YMC-Triart C18 ExRs analytical column (5 µm, 250 × 4.6 mm, YMC Co., Ltd., Kyoto, Japan) equipped with a reversed-phase high-performance liquid chromatography system (RP-HPLC, Dionex Ultimate 3000, Thermo Scientific Inc., Sunnyvale, CA, USA). Sub-fraction 3, which demonstrated the highest yield and scavenging ability among all peptides, was further purified via RP-HPLC using a C18 analytical column with MeOH containing 0.1% formic acid at a flow rate of 1 mL/min for 40 min [32]. Following the method described by Joel et al. [33], a MicrOTOF-Q III mass analyzer (Bruker Daltonics, Bremen, Germany) equipped with electrospray ionization (ESI) was employed to analyze the amino acid sequences in sub-fraction 3.

### 2.5. Experimental Design

Orient Bio (Seongnam, Republic of Korea) provided the 5-week-old male mice (*n* = 66) used in this investigation. Male C57BL/6N mice (*n* = 12, weighing 22–24 g BW) were assigned to the normal (WT) group. Male *db/db* mice (*Lepr^db^*, weighing 36–38 g BW) were homozygous for the diabetes spontaneous mutation, exhibiting chronic hyperglycemia and morbid obesity. Before the experiments, the mice were housed in a temperature-controlled room (22 ± 2 °C) with humidity regulation (55 ± 5%) and subjected to a 12-h light/dark cycle, in adherence to the Guidelines for Care and Use of Laboratory Animals of Chonnam National University. For 6 weeks, the mice were fed a diet consisting of the following ingredients per 1 kg of feed: 2 g of choline bitartrate, 3 g of DL-methionine, 10 g of vitamin mix, 35 g of mineral mix, 50 g of cellulose, 50 g of corn oil, 150 g of corn starch, 200 g of casein lactic acid, and 500 g of sucrose.

Using male *db/db* mice, the acute oral toxicity of MLP hydrolysate was assessed in accordance with the Organization for Economic Cooperation and Development (OECD) guidelines 407 and 425 [34]. Thirty-six male *db/db* mice were split into six groups at random in order to assess the acute toxicity. Six mice (*n* = 6) in each group were kept with free access to food and water. Every day, five different dose levels of MLP hydrolysate solutions (0, 150, 300, 500, 1000, or 2000 mg/kg BW) were prepared and given to the animals orally through gavage in a single dose of 1 mL/100 g BW. Periodically, for the first 0.5, 1, 2, 3, and 4 h following the administration of each MLP hydrolysate dose, as well as once a day for the next 14 days, the general symptoms of the experimental mice were noted. An assessment of the weight changes was carried out on days 0, 7, and 14 following administration. According to the OECD guidelines, we monitored the death, clinical symptoms, and necropsy findings of experimental mice after 14 days of oral administration of the MLP hydrolysate [34].

The *db/db* mice were divided into three groups: the obese *db/db* control (*db/db* CON) and MLP-low (*db/db* MLP-L) or -high (*db/db* MLP-H) groups, each comprising six mice (*n* = 6). The mice in the WT and *db/db* CON groups received an oral dose of 1 mL distilled water (DW), while the *db/db* MLP groups were administered 1 mL of water containing the hydrolysate fraction (≤1 kDa) at a dose of 150 mg/kg BW for the *db/db* MLP-L and 300 mg/kg BW for the *db/db* MLP-H groups once daily (09:00–10:00 a.m.) for 6 weeks. Throughout the study, measurements of the water intake, food consumption, and body weight changes were recorded daily from 8:00 to 9:00 a.m. The food efficiency ratio (FER, %) was calculated: body weight gain (g/day)/food intake (g/day) × 100. Following six weeks of therapy, the mice were sedated by inhaling isoflurane, and blood samples were obtained via heart puncture, from which serum was then extracted. Prior to further investigation, the tissues were collected, weighed, and kept at −80 °C.

### 2.6. Serum Biochemical Analysis

The insulin (INS, #E-EL-M1382), thiobarbituric acid reactive substances (TBARS, #E-BC-K298-M), monocyte chemoattractant protein-1 (MCP-1, #E-UNEL-M0077), tumor necrosis factor-α (TNF-α, #E-UNEL-H0175), and adiponectin (#E-UNEL-M0004) levels were measured using ELISA kits (Elabscience Inc., Houston, TX, USA) [35]. The aspartate aminotransferase (AST, #MAK055), alanine aminotransferase (ALT, #MAK052) activities, total cholesterol (TC, #CS0005), HDL cholesterol, LDL cholesterol (#MAK045), and triglyceride (TG, #MAK266) levels were analyzed using an enzymatic analysis kit following the guidelines of the manufacturer (Sigma-Aldrich, St. Louis, MO, USA). The protein levels were determined using a biuret test (#B3934), albumins were assessed using a BCG kit (#MAK124), and blood urine nitrogen (BUN) was measured using a urease GLDH test kit (#MAS008) (Sigma-Aldrich, St. Louis, MO, USA) [18].

### 2.7. Hepatic Lipid Profile and Antioxidant Enzyme Activities

The liver TC (#CS0005) and TG (#MAK266) levels were analyzed using an ELISA kit (Sigma-Aldrich, St. Louis, MO, USA) following the guidelines of the manufacturer [18]. Antioxidant enzymes, including the glutathione peroxidase (GPx, #ab102530), glutathione S-transferase (GST, #ab65326), glutathione reductase (GR, #ab83461), superoxide dismutase (SOD, #ab65354), catalase (CAT, #ab83464) activities, and glutathione (GSH, #ab65322) levels, were assessed using a colorimetric assay kit and following the instructions of the manufacturer (Biovision Inc., San Francisco, CA, USA) [36].

### 2.8. Histopathological Examinations of Liver and Adipose Tissues

The liver and adipose tissues from the experimental animals were examined histopathologically. After fixing the tissues in 10% (*v*/*v*) formalin, they were embedded in paraffin wax. Dewaxed sections 4–6 µm thick were then produced and stained with hematoxylin (#517282) and eosin (#548243) obtained from Sigma-Aldrich (St. Louis, MO, USA). Pathological changes in the liver tissue and fat tissue were assessed using an optical microscope (Olympus DP70 Microscope, Shinjuku-ku, Tokyo, Japan). The adipocyte area (μm^2^) of the fat tissue was measured using Solution for Automatic Bio-Image Analysis (SABIA) software version 3.0 (EBIOGEN, Seoul, Republic of Korea) [37].

### 2.9. Western Blot Analysis

Using the previously described protocol, the liver tissue was lysed in RIPA buffer (#R0278, Sigma-Aldrich, St. Louis, MO, USA) to isolate the proteins [38]. Subsequently, 40 μg of proteins from each sample was loaded onto the gel. The proteins were separated using SDS-PAGE, and the membrane was blocked for 1 h with 5% skim milk before being incubated with specific primary antibodies overnight at 4 °C. After washing with TBST (Tris-buffered saline with Tween 20), the membranes were incubated with secondary antibodies for 2 h at room temperature. Cell Signaling Technology (Danvers, MA, USA) provided the primary and secondary antibodies for Western blotting analysis. The following antibodies were used: β-actin (# 3700), AMPK (#2532), SREBP-1 (#ab28481), FAS (#3180), and ACC (# 3662). Detection was performed using ECL chemiluminescent (#WP20005, Thermo Scientific, Rockford, IL, USA), and images were captured (iBright^TM^ CL750 Imaging System, Thermo Fisher Scientific Solutions LLC, Seoul, Republic of Korea). The band intensities were analyzed using ImageJ software version 1.53 [18].

### 2.10. qRT-PCR Analysis

RNA was extracted from liver tissue using TRIzol RNA reagent (#15596018, Invitrogen, Carlsbad, CA, USA). The isolated RNA was then used to synthesize cDNA using a cDNA reverse transcription kit (#2708891, Bio-Rad, CA, USA). The qRT-PCR reaction conditions were set to 40 cycles at 94 °C for 15 s and 60 °C for 30 s, with 20 μL of SYBR^®^ Premix Ex Taq (Takara Bio Inc., Shiga, Japan) added to each reaction. The results were normalized against the endogenous GAPDH mRNA signals [39]. Table 1 shows the primer sequences used in this investigation.

### 2.11. Intraperitoneal Glucose Tolerance Test (IPGTT)

Three days before the end of the experiment, MLP pre-treated *db/db* mice were subjected to fasting for 1 day for the IPGTT experiment. The IPGTT test was then performed by injecting 2 g/kg body weight of glucose intraperitoneally. The blood glucose levels were measured at 0, 30, 60, 90, 120, and 180 min after the glucose injection using an Accu-Chek glucose meter (Roche Diagnostics Korea Co., Ltd., Seoul, Republic of Korea). The area under the curve (AUC) was calculated from the data collected during the IPGTT analysis [39].

### 2.12. Statistical Analysis

All parametric data were expressed as means ± SEM. The data were analyzed using IBM SPSS (version 20.0, SPSS Inc., Chicago, IL, USA). Data sets with more than two groups were analyzed using a one-way analysis of variance (ANOVA) followed by Tukey’s post hoc multiple comparison test. Differences were considered significant at *p* < 0.05.

## 3. Results

### 3.1. MLP Enzymatic Hydrolysate Antioxidant, Reducing Power, Viability, and Acute Toxicity Assay

**Figure 1:**
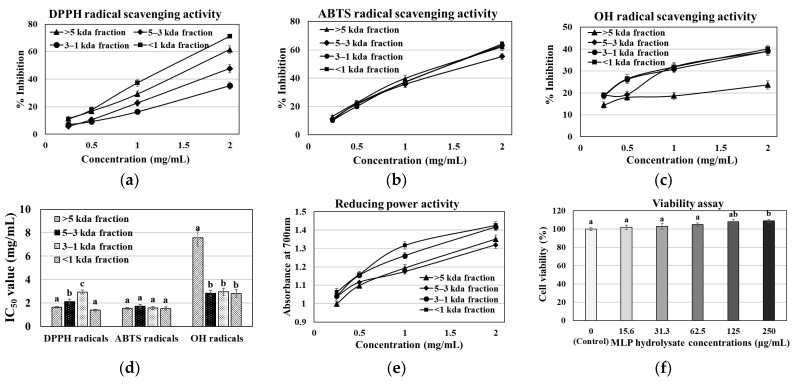
MLP hydrolysate fraction scavenging abilities of (**a**) DPPH radicals, (**b**) ABTS radicals, and (**c**) OH radicals (%); (**d**) IC_50_ values (mg/mL) of all MLP fractions, (**e**) reducing power, and (**f**) cell viability assay. Each value represents the mean ± SEM (*n* = 3). Tukey’s multiple comparison test indicates that values not sharing a common letter are significantly different at *p* < 0.05.

The MLP hydrolysate was obtained from *Meretrix lusoria* using the Protamex hydrolysis enzyme, yielding approximately 64.08%. Figure 1a–e show the MLP hydrolysate fractions (>5, 5–3, 3–1, and ≤1 kDa) that were examined in this study for their capacity to scavenge DPPH, ABTS, and OH radicals. These fractions demonstrated a high scavenging capacity and reducing power, indicated by their lower IC_50_ values. All hydrolysate fractions demonstrated the potential to inhibit DPPH radicals. The hydrolysate ≤1 kDa fraction showed the highest inhibition at 71.24%, followed by the >5 kDa, 5–3 kDa, and 3–1 kDa fractions, with inhibitions of 61.59%, 47.81%, and 35.33%, respectively. Additionally, the ≤1 kDa fraction of the enzymatic hydrolysate showed the greatest ability to scavenge OH and ABTS radicals, with percentages of 40% and 64%, respectively. Compared to larger peptides, this ≤1 fraction may contain a greater number of amino acid groups that are more capable of transferring electrons to free radicals (DPPH, ABTS, and OH). Moreover, the MLP hydrolysate ≤1 kDa fraction demonstrated lower IC_50_ values (1.39–2.8 mg/mL) and a greater scavenging ability for DPPH, ABTS, and OH radicals. Figure 1e shows that this fraction also exhibited a more effective ferric-reducing power than the other fractions (3–1 kDa, 5–3 kDa, and >5 kDa). Nonetheless, the cell viability test using the MLP hydrolysate ≤1 kDa fraction on L6 myoblasts indicated that an MLP fraction up to 250 µg/mL had no impact on cell viability (Figure 1f). Our acute toxicity data show that up to a dosage of 2000 mg/kg BW, none of the MLP hydrolysate-treated groups reported clinical symptoms or death. Furthermore, during the course of the 14-day treatment period, all of the animals remained in excellent health and displayed no alterations in their overall behavior. These findings showed that oral MLP hydrolysate administration at 2000 mg/kg BW did not result in any harmful effects, and the LD_50_ was found to be higher than the dose level examined in this investigation (>2000 mg/kg BW). Owing to their antioxidant and non-toxic effects, our findings suggest that the ≤1 kDa fraction containing bioactive peptides may be employed as a drug delivery agent. This fraction was then further utilized to identify the bioactive peptides using LC-MS/MS analysis. In accordance with earlier publications [16,18,20], we first determined the dosage impact on obese *db/db* mice by selecting the low (150 mg/kg BW) and high (300 mg/kg BW) dose treatments of the MLP fraction (≤1 kDa).

### 3.2. Identification of Bioactive Peptides in ≤1 kDa MLP Enzymatic Hydrolysate

**Figure 2:**
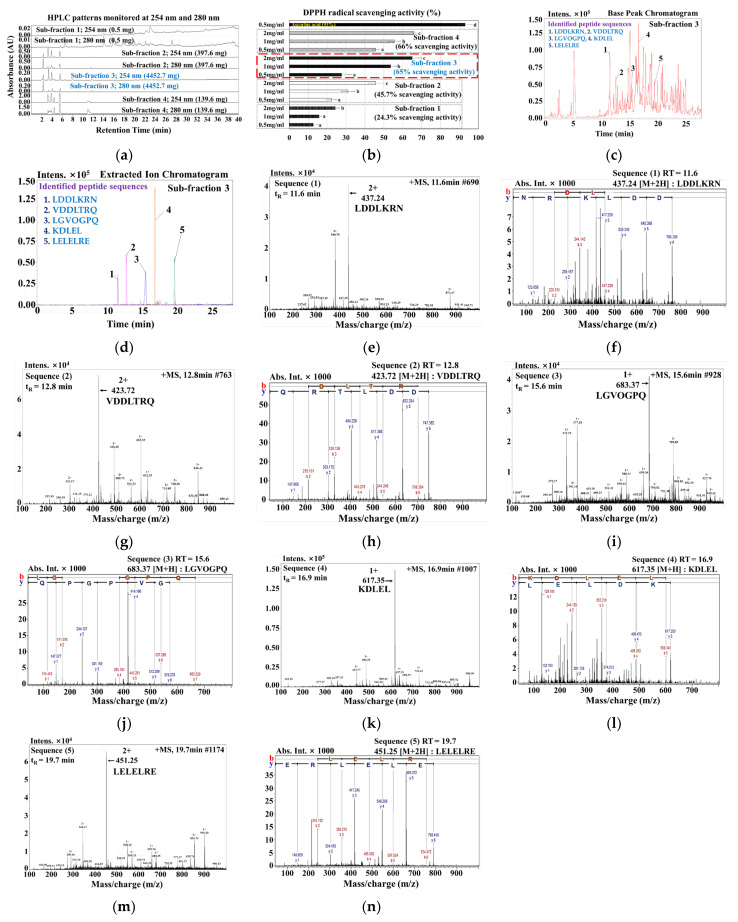
*Meretrix lusoria* hydrolysate fractionation. (**a**) Chromatogram of MLP sub-fractions (1–4) evaluated at 254 and 280 nm using RP-HPLC analysis; (**b**) DPPH radical scavenging activity of four sub-fractions, and highlighted sub-fraction 3 was exhibited high protein yield (4452.7 mg/mL) with effective DPPH radical scavenging ability; (**c**,**d**) Sub-fraction 3 base peak and extracted ion chromatogram from LC-MS analysis, including identified bioactive peptides (1) LDDLKRN, (2) VDDLTRQ, (3) LGVOGPQ, (4) KDLEL and (5) LELELRE; (**e**,**f**) LC-MS chromatogram and spectrum of Sequence 1 (*m/z* 437.24, peak at t_R_ = 11.6 min); (**g**,**h**) LC-MS chromatogram and spectrum of Sequence 2 (*m/z* 423.72, peak at t_R_ = 12.8 min); (**i**,**j**) LC-MS chromatogram and spectrum of Sequence 3 (*m/z* 683.37, peak at t_R_ = 15.6 min); (**k**,**l**) LC-MS chromatogram and spectrum of Sequence 4 (*m/z* 617.35, peak at t_R_ = 16.9 min); (**m**,**n**) LC-MS chromatogram and spectrum of Sequence 5 (*m/z* 451.25, peak at t_R_ = 19.7 min).

The MLP hydrolysate (≤1 kDa) fraction likely contains active peptides, considering its antioxidant ability to scavenge DPPH, ABTS, and OH radicals along with its effective ferric-reducing power. After purification, this active fraction (≤1 kDa) was sub-fractionated into four sections based on molecular size using an HPLC chromatography column Sephadex G-25, with the absorbance measured at 254 and 280 nm (Figure 2a). Among the four isolated fractions, sub-fraction 3 exhibited high protein concentrations (4452.7 mg/mL) and an effective DPPH radical scavenging ability (Figure 2b). Further purification of sub-fraction 3 was performed using a YMC Triart C18 column, and the amino acid sequences were determined using Q-TOF-ESI mass spectroscopy. Figure 2 shows that the five bioactive peptides sequences were identified in peptide sub-fraction 3: Leu-Asp-Asp-Leu-Lys-Arg-Asn (Sequence 1, *m/z* 437.24, t_R_ = 11.6 min), Val-Asp-Asp-Leu-Thr-Arg-Gln (Sequence 2, *m/z* 423.72, t_R_ = 12.8 min), Leu-Gly-Val-Gly-Pro-Gln (Sequence 3, *m/z* 683.37, t_R_ = 15.6 min), Lys-Asp-Leu-Glu-Leu (Sequence 4, *m/z* 617.35, t_R_ = 16.9 min), and Leu-Glu-Leu-Glu-Leu-Arg-Glu (Sequence 5, *m/z* 451.25, t_R_ = 19.7 min) (Figure 2c–n).

### 3.3. Effect of MLP on Body Weight, Lipid Changes, and Serum Biochemical Analysis

After 6 weeks of research, the influence of MLP on obesity in *db/db* mice was investigated. The results show that MLP treatment inhibited weight gain and fat accumulation in the liver and adipose tissues. However, no noticeable differences were observed in the quantity of food consumed between the MLP and *db/db* CON groups, as shown in Table 2. This similarity in the food intake among the obese groups suggests that differences in food consumption did not cause the inhibitory effect of MLP on weight gain and fat tissue accumulation. Moreover, obese *db/db* mice exhibited significantly higher body weights and liver, kidney, mesenteric, epididymal, perirenal, and retroperitoneal fat weights. In addition, the food efficiency ratio (FER) of the *db/db* CON mice was higher than that of the WT group mice, indicating greater weight gain relative to food intake. Conversely, following MLP administration, weight increases in the body and its organs, including epididymal fat weight, were significantly decreased (Table 2).

Given the significant role inflammation plays in obesity-related diseases, we investigated how MLP treatment affects the serum levels of pro-inflammatory cytokines such as TNF-α and MCP-1. Additionally, obesity–diabetes-related indicators, such as adiponectin, insulin, glucose, albumins, proteins, BUN, and TBARS concentrations, were analyzed (Table 3). The results indicate that the *db/db* mice in the *db/db* CON group exhibited significantly higher levels of TNF-α, BUN, and insulin than those in the WT group, indicating typical diabetes symptoms such as insulin resistance. However, after 42 days of MLP treatment, these levels were significantly (*p* < 0.05) inhibited in a dose-responsive manner. Furthermore, serum protein, albumin, MCP-1, and TBARS levels were also significantly suppressed with the high-dose MLP hydrolysate treatment. Despite this, all obese *db/db* mice maintained elevated serum concentrations of adiponectin and blood glucose, likely owing to their high energy density (Table 3).

### 3.4. MLP Treatment Prevented Obesity and Liver Steatosis in db/db Mice

**Figure 3:**
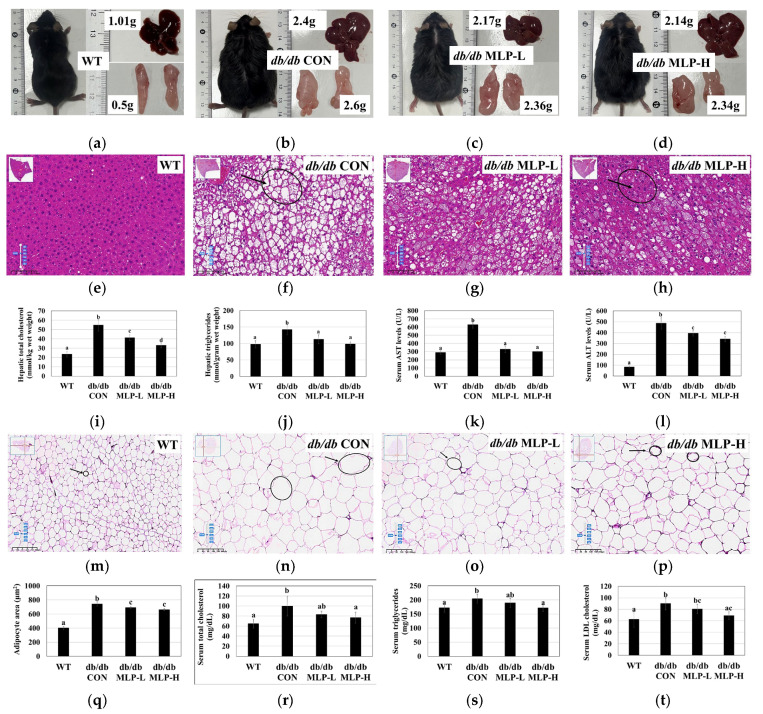
Effect of MLP treatment on obese *db/db* mice. (**a**–**d**) Representative liver and adipose tissues from week 6 of WT, *db/db* CON, and MLP-treated *db/db* mice (**e**–**h**) Representative images of H&E staining at 20× magnification depicting *db/db* CON mouse hepatic steatosis, and MLP therapy in a dose-dependent manner. (**i**–**l**) MLP alleviates hepatic TC and TG contents, including serum AST and ALT levels. (**m**–**p**) Representative images of H&E staining at 10× magnification depicting *db/db* CON mice adipocyte area was increased with fat deposition, MLP therapy reduces the adipocyte area in a dose-dependent manner, and (**q**) adipocyte area. (**r**–**t**) MLP alleviates serum TC, TG, and LDL-cholesterol levels. *db/db* MLP-L (150 mg/kg); *db/db* MLP-H (300 mg/kg). Each value represents the mean ± SEM (*n* = 6). Tukey’s multiple comparison test shows a significant difference (*p* < 0.05) between values that do not share a common letter.

The liver and adipose tissue morphology of obese *db/db* mice treated with MLP hydrolysate was observed using H&E staining. In comparison to those in the WT group, obese *db/db* CON mice exhibited disordered structures with numerous accumulated lipid droplets, showing severe steatosis with vacuolar degeneration in the liver tissue cells. Following MLP hydrolysate intervention, a dose-dependent reduction in fatty acid generation was observed, partially mitigating hepatic cell damage (Figure 3a–h). Additionally, the lipid content analysis revealed significantly increased levels of TC and TG (Figure 3i,j) along with increased liver weights in the obese *db/db* CON group (Figure 3a–d), whereas these levels were significantly decreased in the MLP-treated groups. These findings suggest that MLP can mitigate liver damage and exert a preventive effect on hepatic tissue in obese mice. Elevated levels of AST and ALT in the blood indicate liver inflammation or damage. Significantly reduced serum AST and ALT levels were observed in the MLP-treated groups compared to those in untreated obese *db/db* mice (Figure 3k,l). These findings suggest that MLP hydrolysates may contribute to weight loss and lower blood cholesterol levels.

Furthermore, upon histological examination of epididymal fat tissue, it was observed that obese *db/db* mice in the *db/db* CON group exhibited enlarged white adipocytes with heightened lipid accumulation. In contrast, treatment with MLP led to a significant reduction in the adipocyte size (Figure 3m–p). This indicates that *db/db* mice have a larger adipocyte area (μm^2^) with significant lipid accumulation. However, after 42 days of MLP therapy, this lipid deposition pattern shifted, with numerous smaller molecules replacing the larger lipid droplets. In epididymal tissues, MLP hydrolysate enhanced the overall quantity of small adipocytes while decreasing the number of large-sized adipocytes, as revealed by the morphometric analysis of adipocyte distribution and size (Figure 3m–q). Additionally, an analysis of the serum lipid contents associated with obesity was performed. The obese *db/db* CON group demonstrated significantly higher serum levels of TC, TG, and LDL cholesterol (*p* < 0.05) than the WT group, indicating aberrant lipid metabolism. Following 42 days of oral MLP hydrolysate treatment, the TC, TG, and LDL-cholesterol levels were significantly reduced (*p* < 0.05) compared to the *db/db* CON mice (Figure 3r–t), suggesting that the high-dose MLP treatment had a pronounced effect on lowering blood lipid levels. However, the *db/db* MLP-H group showed higher HDL-cholesterol levels (*p* < 0.05) than the obese *db/db* CON group (Table 3).

### 3.5. Hepatic Antioxidant Enzyme Activities in MLP-Treated Obese Mice

**Figure 4:**
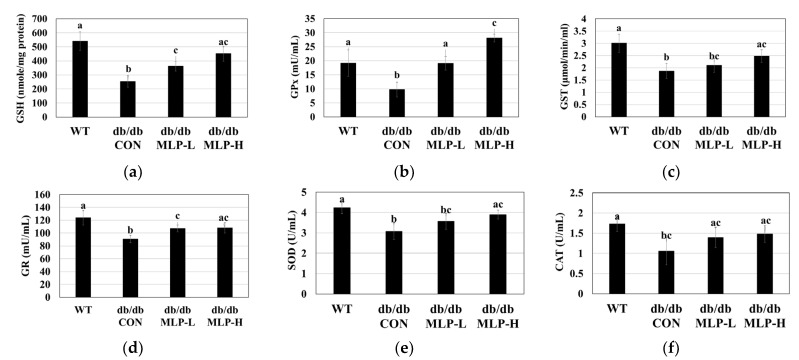
Effect of MLP therapy on antioxidants: (**a**) GSH contents, (**b**) GPx, (**c**) GST, (**d**) GR, (**e**) SOD, and (**f**) CAT enzyme activities in obese *db/db* mice. Each value represents the mean ± SEM (*n* = 6). Tukey’s multiple comparison test shows a significant difference (*p* < 0.05) between values that do not share a common letter.

Figure 4 shows the effect of MLP hydrolysate treatment on the activities of hepatic antioxidant enzymes in obese *db/db* CON mice. When compared to those in the WT group, the obese *db/db* mice demonstrated significantly lower GSH levels, along with reduced activities of antioxidant enzymes, including GPx, GR, GST, SOD, and CAT, in liver tissue homogenates (Figure 4). However, after 42 days of MLP treatment, the hepatic GSH levels and antioxidant enzyme activities were significantly elevated (*p* < 0.05) in a dose-dependent manner.

### 3.6. MLP Regulates Lipid Metabolism by Activating the AMPK Pathway in Obese Mice

**Figure 5:**
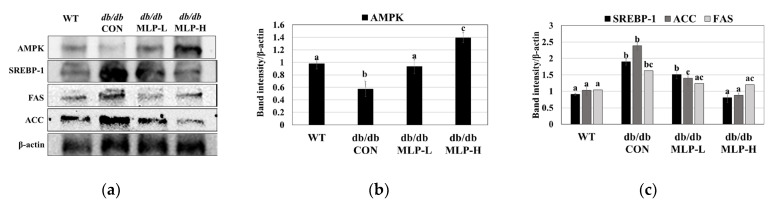
MLP regulated protein expression within the AMPK signaling pathway. (**a**) Proteins AMPK, SREBP-1, FAS, and ACC were identified using a Western blot, and the (**b**) AMPK levels and (**c**) levels of SREBP-1, ACC, and FAS in each group were determined. Each value represents the mean ± SEM (*n* = 4). Tukey’s multiple comparison test shows a significant difference (*p* < 0.05) between values that do not share a common letter.

To unravel the molecular mechanism behind the anti-obesity effect of the MLP hydrolysate fraction, its potential effect on the AMPK pathway in liver tissue obtained from WT and obese *db/db* CON mice was investigated. Our findings revealed that AMPK activity was significantly suppressed in the liver tissue of obese *db/db* CON mice than in WT mice, as indicated by the decreased level of phosphorylated AMPK. Additionally, the expression levels of proteins associated with lipogenesis, including SREBP-1, ACC, and FAS, were elevated (*p* < 0.05) in the *db/db* CON mouse group compared with the WT group. However, following 42 days of MLP therapy, a significant (*p* < 0.05) increase in AMPK activation was observed as well as a a downregulation in the expression of these lipogenic-related proteins (SREBP-1, ACC, and FAS) in the obese *db/db* CON mice (Figure 5a,c).

### 3.7. Lipogenic and Gluconeogenic Enzyme Gene Expressions Using Quantitative RT-PCR

**Figure 6:**
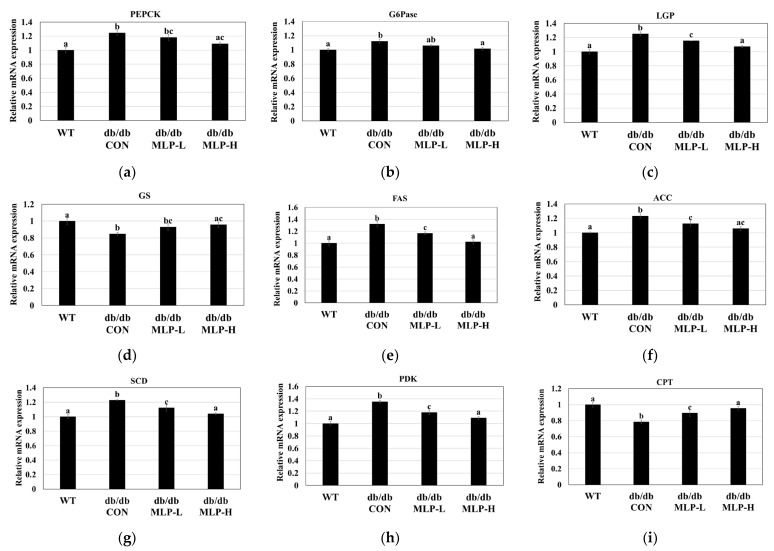
MLP treatment regulated the expressions of genes involved in gluconeogenesis-related metabolic pathways, specifically (**a**) PEPCK, (**b**) G6Pase, (**c**) LGP, (**d**) GS and lipogenic enzymes (**e**) FAS, (**f**) ACC, (**g**) SCD, (**h**) PDK and (**i**) CPT in liver tissues. Each value represents the mean ± SEM (*n* = 4). Tukey’s multiple comparison test shows a significant difference (*p* < 0.05) between values that do not share a common letter.

To unveil the underlying mechanism behind the protective effects of MLP against obesity and diabetes, RT-PCR analysis was performed. Figure 6a–i show that this analysis focused on key enzymes involved in gluconeogenesis (PEPCK, G6Pase, LGP, and GS gene expressions) and lipogenesis (FAS, ACC, SCD, PDK, and CPT gene expressions) in the liver. Our investigations revealed that in obese–diabetic mice of the *db/db* CON group, the transcriptional levels of PEPCK, G6Pase, and GP increased significantly (*p* < 0.05). However, MLP treatment (*db/db* MLP-L and *db/db* MLP-H groups) led to a significant dose-dependent decrease (*p* < 0.05) in the mRNA expressions of these transcription factors (Figure 6a–c). Conversely, the *db/db* CON group demonstrated an inhibition of GS expression, whereas MLP treatment led to a significant increase (Figure 6d; *p* < 0.05). These results showed a reduction in glycogen synthesis in the obese *db/db* CON group compared to the WT group. Additionally, obese *db/db* CON *db/db* mice showed a significant increase (*p* < 0.05) in the mRNA expression levels of the lipogenic enzymes ACC, FAS, SCD, and PDK. However, the administration of MLP led to a dose-dependent, significant reduction (*p* < 0.05) in these levels (Figure 6e–h), indicating the suppression of fat deposition in the liver tissue. Furthermore, while the *db/db* CON group exhibited considerably lower transcriptional levels of CPT compared to the WT group (*p* < 0.05), the MLP groups showed a significant increase in the mRNA expression of these transcription factors compared to the obese *db/db* CON group (Figure 6i; *p* < 0.05).

### 3.8. MLP Improved Glucose Homeostasis in Obese Mice

**Figure 7:**
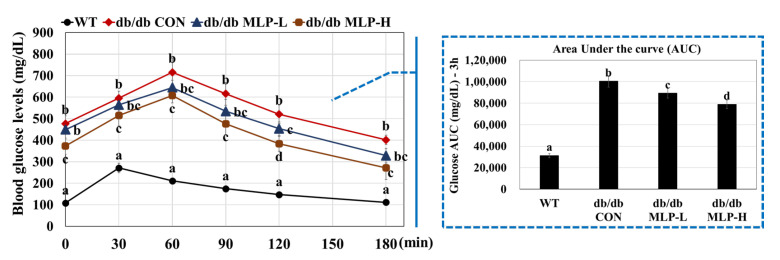
MLP ameliorated glucose homeostasis in *db/db* mice. IPGTT and area under the curve (AUC) of IPGTT. *db/db* MLP-L (150 mg/kg); *db/db* MLP-H (300 mg/kg). Each value represents the mean ± SEM (*n* = 6). Tukey’s multiple comparison test shows a significant difference (*p* < 0.05) between values that do not share a common letter.

MLP was employed to evaluate its anti-diabetic effects in vivo. Given the well-documented association between obesity and T2DM in *db/db* mice owing to a leptin receptor mutation, an IPGTT was conducted at the end of the study to elucidate the metabolic abnormalities associated with obesity (Figure 7). The results showed that MLP pre-treatment led to improved glucose tolerance and enhanced performance. Specifically, compared to the *db/db* CON mice, the MLP pre-treated groups exhibited increased glucose tolerance at 0, 30, 60, 90, 120, and 180 min in a dose-dependent manner. Furthermore, the analysis of the AUC from the IPGTT showed a significant difference between the pre-treated MLP and *db/db* CON groups (*p* < 0.05). These findings suggest that MLP treatment may regulate blood glucose levels by enhancing glucose tolerance in *db/db* mice.

## 4. Discussion

*Meretrix lusoria* is an excellent source of proteins and enzymes, including bioactive peptides, which offer numerous health benefits to consumers [40]. In this study, *Meretrix lusoria* was hydrolyzed using Protamex, and the resulting hydrolysate was fractionated. The antioxidant activity of these fractions was assessed using DPPH, ABTS, and OH radical scavenging assays, along with the reducing power analysis (Figure 1). The *Meretrix lusoria* Protamex (MLP) hydrolysate fraction ≤1 kDa demonstrated a high scavenging ability with a less IC_50_ value (1.39–2.8 mg/mL) for all radicals and exhibited a high reducing power. Therefore, it is important to identify bioactive molecules from the MLP fraction (≤1 kDa) and investigate their anti-obesity effects to combat obesity and related illnesses. The active fraction (≤1 kDa) was further sub-fractionated into four parts using a U-HPLC chromatogram, monitored at 254 and 280 nm, and bioactive compounds were identified from sub-fraction 3 (4452.7 mg/mL). The bioactive peptide sequences LDDLKRN (Leu-Asp-Asp-Leu-Lys-Arg-Asn), VDDLTRQ (Val-Asp-Asp-Leu-Thr-Arg-Gln), LGVOGPQ (Leu-Gly-Val-Gly-Pro-Gln), KDLEL (Lys-Asp-Leu-Glu-Leu), and LELELRE (Leu-Glu-Leu-Glu-Leu-Arg-Glu) were identified using LC-MS/MS analysis. Bioactive peptides, which typically consist of 2 to 20 amino acid residues, can penetrate the intestinal barrier and influence biological functions [31,41,42]. Peptides with higher proportions of hydrophobic amino acids have better lipid solubility and can easily penetrate cell membranes [21]. The authors suggested that the hydrophobic amino acids of the peptides, such as leucine and proline, might contribute to their ability to prevent obesity. Hydrophobic amino acids, including leucine, are necessary for targeting hydrophobic sites. Furthermore, by making lipid-containing peptide molecules more soluble and improving interactions with free radicals, they could prevent lipid peroxidation [43]. Moreover, the peptides are abundant in glutamic acid, leucine, aspartic acid, and lysine, which have a strong ability to scavenge free radicals [18]. The antioxidant peptides possess hydrogen/electron-donating or metal-chelation activities, which allow them to react against free radicals and suppress chain reactions. The peptides identified in this study contain many hydrophobic amino acid residues, including Leu, Val, and Pro. The role of hydrophobic amino acids in radical scavenging and lipid peroxidation prevention has been demonstrated [44]. Moreover, research on animal models and clinical trials published in the literature suggests that bioactive peptides reduce fat accumulation and hepatic steatosis. Additionally, bioactive peptides normalize the glucose-6-phosphate and glycogen levels. This suggests that peptides derived from natural sources have a variety of biological functions, most of which are related to the treatment of human diseases [18,41,42,43].

Studies have shown that *db/db* mice have leptin receptor deficiencies and exhibit characteristics associated with obesity and diabetes at the age of 3 to 4 weeks [7,45]. In these mice, at the age of 4 to 8 weeks, increased excessive blood sugar levels due to a severe depletion of insulin-producing β-cells lead to diabetes and increased gluconeogenesis. In addition, the excessive fat storage and weight gain lead to hyperlipidemia and other obesity-related metabolic issues [46]. This investigation evaluated the ability of MLP to prevent diabetes and obesity in *db/db* mice. The findings indicate that MLP administration regulates the blood glucose levels, lipid contents, and weight gain in obese mice by playing a crucial role in the AMPK pathway. Additionally, the adipocyte cell area significantly increases in obese mice, and obesity is associated not only with larger adipocytes but with an increased number of infiltrating macrophages in adipose tissue. Dysfunctional adipocytes secrete pro-inflammatory cytokines, including MCP-1 and TNF-α. These inflammatory substances can affect the physiology of white adipose tissue (WAT) locally and other organs systemically, leading to insulin resistance [47]. Larger adipocytes show reduced insulin sensitivity, as indicated by impaired insulin-stimulated glucose uptake, which can result in diabetes [48]. Additionally, high serum levels of adiponectin, MCP-1, TNF-α, TBARS, and BUN are primary contributors to obesity-related inflammation in *db/db* mice [18,49]. The current study suggests that 42 days of MLP treatment can reduce pro-inflammatory cytokines, improve adipocyte insulin sensitivity, and decrease body weight.

The histopathological analysis showed that the hepatocytes of obese *db/db* CON mice were enlarged and more disorganized than those of the WT group mice, with intracellular fat vacuoles. This disorganization is owing to the excessive number of fatty acids transferred from adipose tissue to the liver, resulting in a high hepatic load [7]. Fan et al. [50] suggested that fat accumulation is the primary factor causing hepatic inflammation. The histological observation of *db/db* CON mouse fat tissue also showed a greater adipocyte area (μm^2^) with significant fat deposition. Additionally, the obese *db/db* CON mouse group exhibited significantly high levels of hepatic TC and TG levels, elevated liver marker enzymes AST and ALT, and increased serum TC, TG, and LDL-cholesterol levels. However, in the *db/db* CON group, MLP treatment effectively reduced the adipocyte area and body weight by 10–35% in a dose-dependent manner after 42 days. Moreover, MLP alleviated hepatic steatosis and restored the normal arrangement of liver hepatocytes. These findings suggest that MLP could mitigate liver damage in *db/db* mice by increasing serum HDL-C levels and decreasing serum TC, TG, and LDL-C contents, as well as hepatic serum marker enzymes. Dyslipidemia impairs the ability of fat tissue to function as a secretory factor, increasing insulin resistance and leading to diabetes [50]. Conversely, reducing blood TC, TG, and LDL-C levels has been shown to prevent lipid-based cardiovascular disorders [51]. Owing to its diverse activities, including antioxidant, anti-inflammatory, glucose metabolism-enhancing, and cell homeostasis-maintaining properties, MLP is believed to have a protective influence on the liver tissue of obese mice.

Increased fatty acid oxidation and the generation of reactive oxygen species (ROS) result from elevated lipid contents in adipose and hepatic tissues. Oxidative stress is a condition that damages cells or tissues and intensifies the inflammatory response owing to an imbalance between the generation of ROS and the antioxidant enzymes that combat them [52]. Excessive ROS production during oxidative stress can disrupt the intracellular redox balance, accelerating the onset of various illnesses, including diabetes, heart disease, and neurological disorders [21]. The natural antioxidant defense system, which includes GSH, GPx, GST, GR, SOD, and CAT, is crucial for maintaining ROS levels [53]. According to the current findings, MLP therapy in obese *db/db* mice increases the levels of GSH and the liver antioxidant enzymes GPx, GST, GR, SOD, and CAT while lowering the levels of TC, TG, and LDL-C. The peptide length, hydrophobicity, and sequence play major roles in the antioxidant activity demonstrated by MLP hydrolysate [21,54]. Antioxidant peptides typically contain 3–15 amino acid residues [55]. Recent studies have isolated a novel antioxidant peptide (<3 kDa) from pecan protein hydrolysate, Leu-Ala-Tyr-Leu-Gln-Tyr-Thr-Asp-Phe-Glu-Thr-Arg, which exhibits strong antioxidant properties [56]. The pepsin enzymatic hydrolysate of krill provided the antioxidant peptide Ala-Met-Val-Asp-Ala-Ile-Ala-Arg (IC_50_: 0.87), increasing the hepatic antioxidant enzyme activity [57]. The bioactive peptides extracted in this study (<1 kDa) demonstrated potent antioxidant and hydrophobic characteristics. They also revealed the ability to suppress oxidative stress and boost the synthesis of endogenous antioxidant enzymes. Further research is recommended to determine if this unique peptide could be developed into a pharmaceutically potent functional food.

In this study, the expressions of key enzymes involved in gluconeogenesis and hepatic glycolysis, including PEPCK, G6Pase, LGP, and PDK, were examined, as well as the glycogen synthesis enzyme GS. Additionally, the lipid metabolism-related enzymes CPT, ACC, FAS, SREBP, and SCD were analyzed. The results showed an increased protein expression of the lipogenic transcription factor SREBP1 in *db/db* CON mice (Figure 5), stimulating FAS and ACC, thereby enhancing lipogenesis and adipogenesis, leading to increased liver TG and TC levels [58,59]. Elevated SREBP-1 expression can disrupt lipid metabolism in the liver by influencing the glucose/lipid balance [49]. According to Fang et al. [22], SREBP-1 activity is regulated by AMPK expression, and its inhibition may have an anti-hepatic steatosis effect in obese mice. The intracellular energy sensor AMPK plays a role in fat and carbohydrate metabolisms [60,61]. Consequently, when AMPK is activated, ACC becomes inactive, leading to a decrease in cellular malonyl-CoA levels, an endogenous CPT1 inhibitor, which ultimately promotes fatty acid oxidation [2,22]. Additionally, numerous findings revealed that AMPK activation could reduce BW, TG, and TC while increasing insulin sensitivity by downregulating the adipogenic marker enzymes SREBP-1, FAS, and ACC [2,19]. Therefore, targeting the AMPK pathway may offer therapeutic potential for treating obesity, diabetes, and related metabolic disorders by suppressing the levels of lipogenic and gluconeogenesis enzymes, including SREBP-1, FAS, ACC, SCD, PEPCK, G6Pase, LGP, and PDK [12].

**Figure 8:**
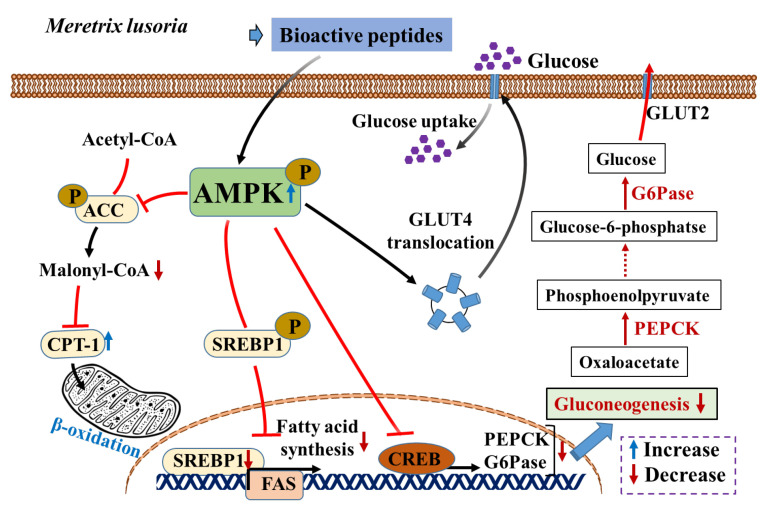
Proposed mechanism for the role of AMPK on lipid and glucose metabolisms in liver tissue. The MLP treatment activates AMPK activity, which leads to downregulated lipogenic enzymes such as ACC and FAS. The results indicate that the AMPK pathway may increase glucose transport as well as fatty acid oxidation.

MLP treatment in obese *db/db* mice led to upregulated AMPK protein expression and downregulated SREBP1, FAS, and ACC levels (Figure 5), resulting in decreased fatty acid synthesis and hepatic lipid accumulation. Additionally, MLP treatment increased the hepatic gene expressions of CPT and GS while decreasing the levels of SCD, PDK, PEPCK, G6Pase, and LGP in obese *db/db* CON mice (Figure 6). Consequently, utilizing MLP hydrolysate to suppress the SREBP1 transcriptional factor involved in lipogenesis, glycolysis, and gluconeogenesis proves to be an effective strategy for preventing obesity, diabetes, and other metabolic disorders (Figure 8). Insulin sensitivity and reduced glucose tolerance are prevalent coexisting conditions in obesity and diabetes [62]. Our IPGTT and AUC results showed that the blood glucose levels reduced by 11–21% after 3 h in MLP-treated groups in a dose-dependent manner, indicating that MLP treatment could not only reduce body weight but also ameliorate impaired glucose tolerance in obese *db/db* CON mice. These findings reveal that MLP therapy can reduce gluconeogenesis by activating the AMPK system and increasing peripheral glucose absorption [63]. Conversely, activated AMPK may enhance glucose utilization by promoting GLUT4 production and its translocation from intracellular storage vesicles to cell membranes in skeletal muscle and adipose tissues [62]. Our findings indicate that MLP hydrolysates containing bioactive peptides can activate AMPK, leading to decreased lipid levels and improved insulin resistance [12]. Therefore, utilizing MLP therapy as an AMPK activator has shown benefits for lipid-related disorders. 

## 5. Conclusions

Our results show that bioactive peptides from MLP hydrolysate alleviate obesity and diabetes and are associated with metabolic complications. MLP administration effectively reduces lipid accumulation in the liver and adipose tissues. The findings indicate that MLP is most effective in promoting weight reduction by regulating the expressions of SREBP-1 transcriptional factors and modulating the actions of lipogenesis and TG synthesis-related enzymes, including ACC, FAS, SCD, and PDK, among others. The activation of the AMPK signaling pathway mediates these effects, leading to the suppression of fat formation in the liver and adipose tissues. Our research demonstrates that bioactive peptides derived from MLP alter AMPK-related molecular pathways, exerting a beneficial effect on metabolic illnesses, including obesity and diabetes. However, further studies are required to identify any adverse reactions from long-term MLP hydrolysate treatment, including at higher dosages, on obese and diabetic mice.

## Figures and Tables

**Table 1:** List of primers used in this study for gene expression analysis.

Gene ^1^	Forward Primer	Reverse Primer
ACC	GACGTTCGCCATAACCAAGT	CTGCAGGTTCTCAATGCAAA
G6Pase	ATGACTTTGGGATCCAGTCG	TGGAACCAGATGGGAAAGAG
LGP	CCAGAGTGCTCTACCCCAAT	CCACAAAGTACTCCTGTTTCAGC
GS	GACACTGAGCAGGGCTTTTC	GGGCCTGGGATACTTAAAGC
PEPCK	CTGGCACCTCAGTGAAGACA	TCGATGCCTTCCCAGTAAAC
FAS	CCCTTGATGAAGAGGGATCA	ACTCCACAGGTGGGAACAAG
CPT	GAGCCACGAAGCCCTCAAACACAT	GCTGTACAACATGGGCTTCCGACCTG
PDK	ATCTAACATCGCCAGAATTAAACC	GGAACGTACACAATGTGGATTG`
SCD	AGCTGGTGATGTTCCAGAGG	GTGGGCAGGATGAAGCAC

^1^ Acetyl-CoA carboxylase (ACC), glucose 6-phosphatase (G6Pase), liver glycogen phosphorylase (LGP), glycogen synthase (GS), phosphoenolpyruvate carboxykinase (PEPCK), fatty acid synthase (FAS), carnitine palmitoyltransferase (CPT), pyruvate dehydrogenase kinase (PDK), and stearoyl-CoA desaturase (SCD).

**Table 2:** Effect of MLP treatment on *db/db* mouse body and organs weight changes, including fat contents.

Measurement	WT	*db/db* CON	*db/db* MLP-L	*db/db* MLP-H
Initial BW (g)	23.67 ± 0.94 ^a^	36.17 ± 1.07 ^b^	36.67 ± 1.37 ^b^	36.67 ± 1.37 ^b^
Water intake (ml/day)	5.60 ± 0.07 ^a^	17.99 ± 2.63 ^b^	17.25 ± 3.29 ^b^	17.49 ± 3.00 ^b^
Food intake (g/day)	2.46 ± 0.20 ^a^	4.67 ± 0.21 ^b^	4.62 ± 0.48 ^b^	4.56 ± 0.34 ^b^
FER (%)	1.40 ± 0.62 ^a^	3.16 ± 1.05 ^bc^	2.68 ± 0.57 ^ac^	2.16 ± 1.01 ^ab^
BW gain (g/day)	0.03 ± 0.01 ^a^	0.14 ± 0.05 ^b^	0.13 ± 0.02 ^b^	0.10 ± 0.05 ^c^
Heart (g)	0.110 ± 0.009 ^a^	0.132 ± 0.004 ^b^	0.121 ± 0.010 ^ab^	0.117 ± 0.010 ^ab^
Kidney (g)	0.290 ± 0.024 ^a^	0.404 ± 0.023 ^b^	0.363 ± 0.017 ^b^	0.359 ± 0.030 ^b^
Liver (g)	1.013 ± 0.083 ^a^	2.359 ± 0.096 ^b^	2.174 ± 0.201 ^b^	2.145 ± 0.158 ^b^
Gastrocnemius muscle (g)	0.270 ± 0.013 ^a^	0.195 ± 0.030 ^b^	0.220 ± 0.035 ^ab^	0.215 ± 0.031 ^ab^
Mesenteric fat (g)	0.354 ± 0.013 ^a^	1.751 ± 0.153 ^b^	1.690 ± 0.186 ^b^	1.482 ± 0.101 ^b^
Fat retroperitoneal (g)	0.120 ± 0.019 ^a^	0.519 ± 0.076 ^b^	0.454 ± 0.037 ^b^	0.439 ± 0.065 ^b^
Fat epididymal (g)	0.493 ± 0.068 ^a^	2.5801 ± 0.051 ^b^	2.369 ± 0.153 ^b^	2.346 ± 0.103 ^b^
Perirenal fat (g)	0.060 ± 0.012 ^a^	0.531 ± 0.037 ^b^	0.456 ± 0.104 ^bc^	0.352 ± 0.075 ^c^
Fat total (g)	1.026 ± 0.089 ^a^	5.382 ± 0.200 ^b^	4.969 ± 0.358 ^bc^	4.619 ± 0.192 ^c^

Each value represents the mean ± SEM (*n* = 6). Tukey’s multiple comparison test shows a significant difference (*p* < 0.05) between values that do not share a common letter.

**Table 3:** Effect of MLP treatment on serum biochemical markers associated with obesity and diabetes.

Measurement	WT	*db/db* CON	*db/db* MLP-L	*db/db* MLP-H
Initial fasting blood glucose (mg/dL)	120.00 ± 14.04 ^a^	170.50 ± 17.70 ^b^	170.33 ± 16.79 ^b^	170.83 ± 17.72 ^b^
Fasting blood glucose at the end of treatment (mg/dL)	108.16 ± 12.74 ^a^	476.66 ± 36.99 ^b^	449.16 ± 43.69 ^b^	372.5 ± 38.23 ^c^
MCP-1 (pg/mL)	193.47 ± 51.96 ^a^	963.75 ± 52.43 ^b^	920.83 ± 45.64 ^b^	734.58 ± 33.97 ^c^
TNF-α (pg/mL)	372.05 ± 7.45 ^a^	606.35 ± 7.45 ^b^	501.96 ± 18.88 ^c^	433.82 ± 18.49 ^d^
Adiponectin (ng/mL)	1.22 ± 0.07 ^a^	2.67 ± 0.26 ^b^	2.59 ± 0.09 ^b^	2.44 ± 0.13 ^b^
TBARS (μmol/L)	68.52 ± 6.05 ^a^	90.68 ± 5.95 ^b^	81.03 ± 5.84 ^bc^	74.62 ± 7.74 ^ac^
HDL-cholesterol (mg/dL)	166.50 ± 13.50 ^a^	239.00 ± 18.79 ^c^	260.00 ± 14.56 ^b^	262.50 ± 17.04 ^b^
Insulin (ng/mL)	1.30 ± 0.05 ^a^	3.05 ± 0.04 ^b^	2.85 ± 0.03 ^c^	2.63 ± 0.05 ^d^
Total protein (g/dL)	5.58 ± 0.73 ^a^	6.83 ± 0.40 ^b^	6.65 ± 0.16 ^b^	5.70 ± 0.21 ^a^
Albumin (g/dL)	3.75 ± 0.52 ^a^	4.63 ± 0.28 ^b^	4.62 ± 0.11 ^b^	4.02 ± 0.16 ^a^
BUN (mg/dL)	16.50 ± 1.26 ^a^	28.33 ± 4.71 ^b^	20.33 ± 1.60 ^a^	18.33 ± 3.20 ^a^

Each value represents the mean ± SEM (*n* = 6). Tukey’s multiple comparison test indicates that values not sharing a common letter are significantly different at *p* < 0.05.

## Data Availability

Data is contained within the article.
